# First human safety and effectiveness study of defibrillation with a novel patch wearable cardioverter-defibrillator

**DOI:** 10.1093/europace/euae189

**Published:** 2024-07-13

**Authors:** Milan Chovanec, Jan Petrů, Pavel Hála, Stepan Kralovec, Anjali B Thakkar, Kiran Mathews, Maarten Dinger, Steven Ullery, Zubin J Eapen, Uday N Kumar, Petr Neužil

**Affiliations:** Department of Cardiology, Na Homolce Hospital, Prague, Czech Republic; Department of Cardiology, Na Homolce Hospital, Prague, Czech Republic; Department of Cardiology, Na Homolce Hospital, Prague, Czech Republic; Department of Cardiology, Na Homolce Hospital, Prague, Czech Republic; Division of Cardiology, Department of Medicine, University of California San Francisco, 505 Parnassus Avenue, San Francisco, CA 94143, USA; Element Science, Inc., Redwood City, CA, USA; Element Science, Inc., Redwood City, CA, USA; North American Science Associates, Walnut Creek, CA, USA; Element Science, Inc., Redwood City, CA, USA; Element Science, Inc., Redwood City, CA, USA; Department of Cardiology, Na Homolce Hospital, Prague, Czech Republic

**Keywords:** Wearable cardioverter-defibrillator, Sudden cardiac arrest, Ventricular arrhythmia

## Abstract

**Aims:**

Wearable cardioverter-defibrillators (WCDs) are indicated in patients at risk of sudden cardiac arrest who are not immediate candidates for implantable defibrillator therapy. Limitations of existing WCDs include poor compliance and high false alarm rates. The Jewel is a novel patch-WCD (P-WCD) that addresses these limitations with an adhesive-based design for near-continuous wear and a machine learning algorithm designed to minimize inappropriate detections. This was a first-in-human study of the Jewel P-WCD conducted in an electrophysiology (EP) lab to determine the safety and effectiveness of the device in terminating ventricular tachycardia/ventricular fibrillation (VT/VF) with a single shock. The aim was to evaluate the safety and effectiveness of terminating VT/VF with a single shock using the Jewel P-WCD.

**Methods and results:**

This was a first-in-human, prospective, single-arm, single-centre study in patients scheduled for an EP procedure in which VT/VF was expected to either spontaneously occur or be induced. The Jewel P-WCD was placed on consented patients; upon confirmation of VT/VF, a single shock (150 J) was delivered via the device. A group sequential design and Pocock alpha spending function was used to measure the observed proportion of successful VT/VF single-shock terminations. The endpoint was achieved if the lower confidence limit exceeded the performance goal of 62%, using a one-sided lower 97.4% exact confidence bound. Of 18 eligible subjects, 16 (88.9%, 97.4% confidence bound: 65.4%) were successfully defibrillated with a single shock, exceeding the primary endpoint performance goal with no adverse events.

**Conclusion:**

This first-in-human evaluation of the Jewel P-WCD demonstrated the safety and effectiveness of terminating VT/VF.

**Clinical Trial Registration:**

URL: https://clinicaltrials.gov/; Unique identifier: NCT05490459

## Graphical Abstract

**Graphical Abstract:**
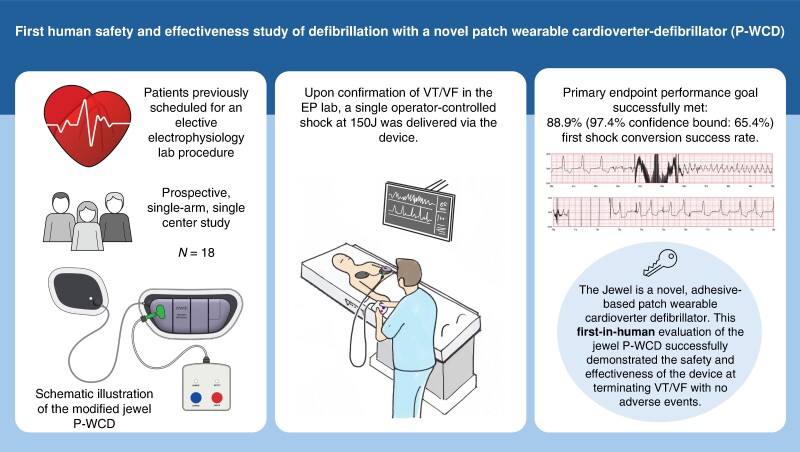

**What’s new?**

- The Jewel is a novel patch wearable cardioverter-defibrillator (P-WCD) with an adhesive-based design for near-continuous compliance and a machine learning algorithm designed to minimize inappropriate detections.
- The results of this first-in-human study demonstrate the safety and effectiveness of terminating ventricular tachycardia/ventricular fibrillation (VT/VF) using a standard defibrillation waveform with a novel delivery system designed to improve patient compliance. This could have implications in the management of patients at increased risk of sudden cardiac arrest who either temporarily do not qualify for an implantable cardioverter-defibrillators (ICD) or have long-term contraindications to ICD therapy, as the ability of WCDs to terminate VT/VF is dependent on patient compliance.

## Introduction

Sudden cardiac arrest (SCA) is the leading cause of natural death in the USA, accounting for ∼325 000 deaths each year.^1,2^ Implantable cardioverter-defibrillators (ICDs) can improve outcomes in many patient groups at increased risk of SCA.^3–7^ However, during temporary periods of elevated risk when an ICD is not present—such as in the 40 days after myocardial infarction—wearable cardioverter-defibrillators (WCDs) can provide protection against SCA until an ICD can be implanted.^8^

The major limitation with currently available WCDs is low patient compliance associated with arrhythmic death.^9,10^ Element Science, Inc., has developed the Jewel patch-WCD (P-WCD; ‘Jewel’) to improve the comfort and wearability of a WCD by focusing on a patient-centric design. The Jewel is a novel, low-profile, water-resistant P-WCD designed to enhance compliance by optimizing comfort, reducing maintenance and patient involvement, and allowing use during most activities, including showering, sleeping, and moderate exercise. The ECG signal is monitored through continuously adhered electrodes designed to minimize motion artefact and to deliver energy uniformly when shocks are required. The Jewel is capable of delivering a salvo of one shock at 150 J and up to four additional shocks at 162 J each per event and uses a biphasic truncated exponential (BTE) waveform that is adjusted based on the transthoracic impedance of the patient.

This study describes the first-in-human experience of terminating ventricular tachycardia (VT) or ventricular fibrillation (VF) with the Jewel P-WCD. Demonstrating defibrillation effectiveness is limited by the low incidence of VT/VF among ambulatory patients at risk for SCA. Therefore, this study was conducted in a population of individuals who were already undergoing an electrophysiology (EP) lab study in which they would be induced into VT/VF. To optimize for patient safety, this study was conducted using a modified version of the Jewel P-WCD in which shock delivery was operator-controlled to minimize the time that a patient would spend in VT/VF. The safety and effectiveness of the Jewel P-WCD designed for commercial use were subsequently evaluated in an ambulatory (‘real-world’) setting in the intended use population in the Jewel IDE Study (NCT05201495). This study successfully met primary and secondary endpoints demonstrating safety and effectiveness over the duration of real-world prescription lengths, and multiple successful conversions were observed.^11^

## Methods

### Study design

A prospective, single-arm, single-centre, non-randomized, non-blinded feasibility study (URL, https://www.clinicaltrials.gov/; unique identifier, NCT05490459) was conducted in individuals already scheduled for a standard EP procedure in which life-threatening VT or VF was expected to either spontaneously occur or be induced. All participants were recruited from Na Homolce Hospital, Prague, in the European Union. This study used a historical comparator based on randomized clinical trial data of the first shock success rate of a commercially available wearable defibrillator (LifeVest) as its control. This allowed for the study to use a pre-specified statistical performance goal for defibrillation effectiveness with a single shock. Because this is a single-arm study with a historical control, no randomization or additional comparison groups were required.

This study was conducted in accordance with the US Code of Federal Regulations applicable to clinical studies, EN ISO 14155:2020, applicable local regulations, and the ethical principles that have their origin in the Declaration of Helsinki. The study and amendments were reviewed by the Na Homolce Hospital Ethics Committee and the Czech Republic Competent Authority. Written informed consent was obtained from all subjects after being screened for study eligibility and prior to enrolment into the trial.

### Participants

Adult participants who were already scheduled for a standard EP clinical procedure where life-threatening VT or VF may spontaneously occur or may be induced were recruited. Eligibility criteria are summarized in *Table 1*. Key exclusion criteria included left ventricular ejection fraction (LVEF) < 20%, recent diagnosis of heart failure, NYHA Class IV heart failure, unstable angina, recent amiodarone use, and atrial fibrillation with a contraindication to anticoagulation or improper anticoagulation management.

**Table 1:** Eligibility criteria for participation in the EP lab study

Inclusion criteria
Adult patients (at least 18 years of age) of both genders
Already scheduled for a standard EP clinical procedure where life-threatening VT or VF may spontaneously occur or may be induced.
**Exclusion criteria**
Subjects who may require sterile access to the right upper pectoral or lower left torso regions during the planned EP procedure.
Subjects who have taken amiodarone in the past 3 months.
Subjects with an existing unipolar pacemaker.
Subjects who exhibit a LVEF < 20% (as assessed by techniques such as echocardiography, magnetic resonance imaging, or radionuclide angiography within the last 6 months).
Subjects who have been diagnosed with heart failure (Class IV) or experienced an acute heart failure exacerbation within the previous 30 days.
Subjects who exhibit unstable angina.
Subjects with atrial fibrillation with contraindication to anticoagulation or improper anticoagulation management.
Subjects who are participating in an investigational study of a drug, biologic, or device not currently approved for marketing.
Subjects who are allergic to or have had a known adverse reaction to medical adhesives.
Subjects who have active skin breakdown, erythema, or other signs of infection in the pectoral or torso regions where the study device is applied.
Subjects with a lower abdomen circumference of <68.5 cm or >142 cm.
Females who are pregnant, breastfeeding, or planning to be pregnant in the next 12 months.
Subjects who cannot provide or have diminished capacity to provide informed consent.
Any condition that an investigator believes would interfere with the intent of the study or is not in the best interest of the patient.
Any patient that according to the Declaration of Helsinki is unsuitable for enrolment.

### Description of the modified Jewel patch wearable cardioverter-defibrillator

**Figure 1:**
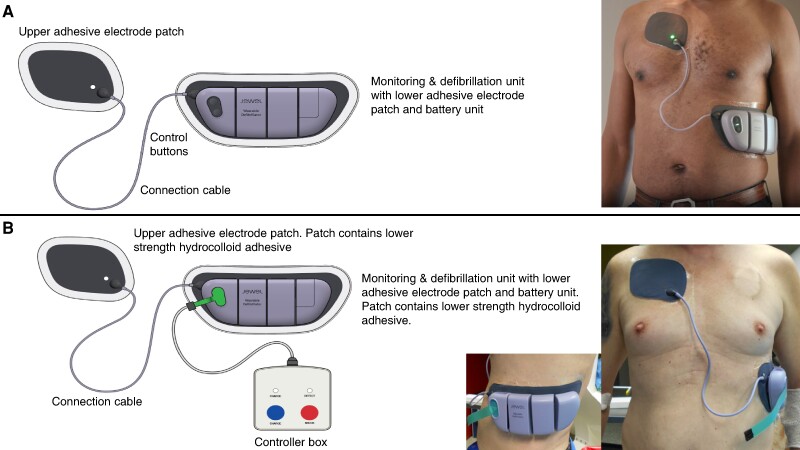
Schematic diagrams of the Jewel P-WCD and Jewel EP lab device. (*A*) Schematic diagram of the commercial-use Jewel P-WCD components and photo of the Jewel P-WCD applied to a patient’s body. (*B*) Schematic diagram of the manually operated Jewel P-WCD components (used in this study) including operator shock control and photo of the modified Jewel P-WCD applied to a patient’s body.

The novel patch design of the Jewel allows the device to be worn continuously and comfortably for up to 8 days, including during daily activities such as showering and sleeping. Schematics of the Jewel P-WCD designed for commercial use and the modified Jewel P-WCD intended for defibrillation testing in the EP lab setting are shown in *Figure* *1A* and *B*, respectively.

The Jewel consists of a disposable upper adhesive electrode patch, a reusable monitoring and defibrillation unit (MDU) attached to a disposable lower adhesive electrode patch with permanently attached disposable battery unit and the attached connection cable (*Figure 1A*). The two adhesive patches and MDU are worn continuously for up to 8 days. The Jewel monitors a patient’s ECG through two defibrillation electrodes (with backup monitoring via two additional electrodes) located on the upper and lower adhesive electrode patches. The product designed for commercial use employs a proprietary machine learning–based algorithm to detect and classify fast VT or VF that is deemed to be life-threatening (‘shockable’) vs. cardiac rhythms that are non–life-threatening (‘non-shockable’). The Jewel algorithm will monitor a shockable rhythm for 24 s. If a shockable rhythm persists after this time period, the Jewel firmware initiates the charge cycle of the capacitors, and in parallel, the Jewel will initiate an alarm sequence to alert the patient that the device is preparing to deliver a shock. If the patient is conscious, the patient is instructed to press the control buttons to stop the alarms and prevent the delivery of the shock. If the patient does not respond, and the shockable rhythm continues, the Jewel will continue to alarm the patient and will give a verbal warning to bystanders that a shock will be delivered. The Jewel delivers the initial therapeutic shock with a fixed energy of 150 J with a BTE defibrillation waveform consistent with other well-tested waveforms using a constant energy pulse that is adjusted based on the transthoracic impedance of the patient at the time of therapy delivery. Using a cardioversion algorithm, the Jewel will attempt to cardiovert the rhythm and synchronize the shock. After the initial shock of 150 J, if the Jewel continues to detect a shockable rhythm, the Jewel will re-initiate the alarm sequence and continue to deliver a salvo of up to four additional shocks of 162 J, totalling five consecutive shocks (150, 162, 162, 162, and 162 J).

The modified Jewel P-WCD used in this study has an additional controller box (*Figure 1B*). The controller box allows the operator to manually charge the Jewel capacitors as well as manually administer a defibrillation shock when the operator has determined that the presenting rhythm warrants defibrillation. This minimizes the safety risk to both the patient by limiting VT or VF to a relatively short time period of <20–30 s and the medical personnel in the EP lab environment to ensure that shock delivery only occurs when all bystanders are clear of the patient. Because of this, the machine learning–based VT/VF algorithm and synchronized cardioversion algorithm which are integrated into the Jewel P-WCD designed for commercial use were not used in this study. Additionally, the modified Jewel P-WCD used in this study contains a lower strength hydrocolloid adhesive on the patches as patients are only expected to wear the device for <24 h. All other elements of the Jewel P-WCD used in this study, including the design of the patches worn, hydrogel used for defibrillation, ECG electrodes, the amount of energy delivered by the initial shock, and the defibrillation waveform are the same as in the Jewel P-WCD designed for commercial use.

### Experimental protocol

Prior to undergoing the planned procedure, the Jewel P-WCD was applied to subjects using the same application process that a patient would complete during commercial use. Hospital-supplied rescue defibrillator pads (Medtronic Lifepak 20) were placed on the subject in an anterior–posterior position, ensuring a 1–2 cm margin between the defibrillation pads and the adhesive electrode patches. A hospital-supplied ECG was used per standard hospital procedure. All patients underwent moderate sedation per usual hospital procedural protocol. The investigator proceeded with the planned EP clinical procedure and heart rhythm monitoring per standard of care at the hospital facility. Ventricular tachycardia/ventricular fibrillation was induced by burst pacing (Micropace EPS-200 Cardiac Stimulator 3.21) from the right ventricular apex area with a quadripolar Supreme™ EP catheter (Abbott, 6F, 2.0 mm tip), advanced through Fast-Cath™ haemostasis introducer 7F (Abbott) via right femoral venous access. Prior to the onset of life-threatening VT or VF, the hospital staff prepared the Jewel P-WCD by initiating the device charge cycle. For safety, the rescue defibrillator was also prepared for use in case the Jewel P-WCD did not deliver an electrical shock or failed to terminate VT/VF using a single defibrillation shock.

Throughout the procedure, ECG signals were continuously monitored both by the Jewel P-WCD and by hospital staff. Once VT/VF occurred or was induced and confirmed by the electrophysiologist performing the study, the Jewel P-WCD was used to deliver a single defibrillation shock to terminate the rhythm. If a single defibrillation shock delivered through the Jewel P-WCD did not successfully terminate the VT/VF, the rescue defibrillator was used to deliver subsequent defibrillation shock(s). While the Jewel P-WCD is capable of delivering four additional shocks per event at a higher energy of 162 J, only the success of the first shock at 150 J was tested so as to not expose patients to prolonged VT/VF. The Jewel P-WCD was used in appropriate cases as determined by physician judgement.

The subjects were recovered following normal hospital protocol. The study did not require follow-up after the completion of the EP Lab procedure except in the event of adverse events in which case the subject would be followed until resolution. Laboratory measurements were not analysed for this study.

### Primary efficacy endpoint

The primary efficacy endpoint was the percentage of successful single-shock terminations of life-threatening VT or VF (first shock conversion success rate), as determined by the treating physician. The first shock conversion success rate endpoint was determined based on clinical trial results studies of a predicate WCD—the ZOLL LifeVest. This was to ensure that the performance of the Jewel was compared in this study to the efficacy of commercially available WCDs. The VEST trial was a randomized controlled trial of the real-world performance of the ZOLL LifeVest in over 2300 subjects.^9^ Of the 21 appropriate shocks, 13 were converted with the first shock and 8 required ≥2 shocks, translating into a 62% first shock conversion success rate. Based on these results, the primary efficacy endpoint would be achieved if the lower confidence limit exceeded the performance goal of 62% using a one-sided exact lower 97.4% confidence bound at one of three testing points.^9^

Any adverse events observed during the acute procedure were tabulated and presented along with the duration, severity, and relatedness to the modified Jewel P-WCD.

### Sample size and statistical methods

The sample size was based on a group sequential design measuring the observed proportion of successful life-threatening VT or VF terminations. The group sequential design used a Pocock alpha spending function and allowed for testing for conversion success after 12, 18, and 24 subjects were enrolled, as described below^12^:

1. If after attempting to convert the first 12 subjects, all subjects have been successfully converted (100% conversion rate), the trial would be stopped and deemed a success. Otherwise, the study would proceed to the next stopping point.
2. If conversion is <100% after 12 total subjects, the study would enrol six additional subjects for a total of 18 subjects. If 17 out of the 18 were successfully converted (94.4% conversion rate), or if 16 out of the 18 were successfully converted (88.0% conversion rate), the study would be stopped and deemed a success. Otherwise, the study would continue to a final stopping point.
3. The study would then enrol the final six subjects for a total of 24 subjects. If after attempting to convert 24 subjects, 21 of the 24 were successful (87.5% conversion rate), the trial would be deemed a success.

The design controlled the overall type I error rate at 5%. In line with the Pocock alpha spending function, a one-sided lower 97.4% exact confidence bound was calculated using the exact binomial (Clopper–Pearson) method at each testing point. The adaptive study design allowed for continuous enrolment of eligible subjects until the sample size was met for the treatment analysis per the statistical plan.

## Results

Between 27 November 2018 and 7 October 2021, 20 patients were enrolled. All patients were already scheduled for a standard clinical EP procedure. Two subjects were excluded from the final per-protocol analysis due to amiodarone use in the 3 months prior to enrolment, an exclusion criterion.

### Demographic and clinical characteristics

Demographic and clinical characteristics of all individuals enrolled in the study are presented in *Table 2*. Among the 18 participants included in the per-protocol analysis, mean age was 63.8 years, 83% were male, and 100% were white. Patients with a wide range of body mass indexes (BMIs) were included in this study (mean 28.3, range 20.2–36.9). Mean LVEF was 34% (range 20–55%). Two patients had a history of a recent myocardial infarction; 3 patients had a history of SCA; 2 patients had a history of VT; and 5 patients had an ICD explanted. The most prevalent comorbidities were hypertension (56%) and prior or current tobacco use (61%).

**Table 2:** Demographic and clinical characteristics of participants enrolled in the EP lab study

Demographic characteristic	Per-protocol subject, *n* = 18
Male (%)	15/18 (83%)
Age, years (range)	63.8 (28–80)
Race, white (%)	18/18 (100%)
Height, cm (range)	176.8 (160–190)
Weight, kg (range)	88.9 (58–129)
BMI (range)	28.3 (20.2–36.9)

Clinical characteristic	*n* (%)
LVEF, % (range)	34 (20–55)
Recent myocardial infarction (%)	2/18 (11%)
Recent coronary artery bypass graft (%)	0/18 (0%)
Unstable angina (% of total)	0/18 (0%)
NYHA Class IV chronic heart failure (%)	0/18 (0%)
SCA (%)	3/18 (17%)
ICD explanted (%)	5/18 (28%)
Hypertension (%)	10/18 (56%)
Prior or current tobacco use (%)	11/18 (61%)
Non-sustained ventricular tachycardia (%)	1/18 (6%)
VT (%)	2/18 (11%)
Diabetes (%)	5/18 (28%)
Atrial fibrillation (%)	3/18 (17%)

### Results of defibrillation testing with the Jewel electrophysiology lab device

Of the 18 subjects included in the per-protocol analysis, 16 (88.9%) were successfully converted back to their baseline rhythm after a single defibrillation shock from the Jewel P-WCD. Two subjects were not successfully converted back to their baseline rhythm with the one allowed defibrillation shock from the Jewel P-WCD and were successfully converted with a single shock using the hospital-provided defibrillator. Based on device data from all participants in the study, the mean energy delivered was 150.7 J ± 0.39% which was below the energy delivery variance threshold of 150 J ± 15%.

The first shock conversion success rate of the Jewel P-WCD observed in this study at the second predetermined early stopping point was 88.9% with a one-sided lower 97.4% exact confidence bound of 65.4%. This exceeded the performance goal of 62% first shock conversion success rate pre-specified in the study protocol. The study was stopped after enrolment of 20 patients as the pre-specified conversion success rate endpoint was met. No adverse events, including no cutaneous adverse events such as burns or rashes, and no deaths were reported in any of the 18 subjects for the duration of their participation in the study.

## Discussion

The results of this first-in-human study demonstrate the safety and effectiveness of terminating VT/VF using the defibrillation waveform and delivery system of a novel P-WCD. The ability of WCDs to terminate VT/VF is dependent on patient compliance. As such, a new technology designed to enhance patient usability and convenience with comparable therapeutic efficacy would be beneficial. This is the first study to our knowledge of a patch wearable device with defibrillation capability and demonstrates that the Jewel P-WCD could be a viable alternative to the currently available WCDs.

Prior studies have demonstrated the safety and effectiveness of existing WCDs in terminating life-threatening VT/VF.^13–16^ In the VEST trial, the first shock conversion success rate of the ZOLL LifeVest was 62%^9^ and in the ACE-CONVERT study, the first shock conversion success rate of the Kestra ASSURE WCD was 84.6% in the intention-to-treat analysis and 83.3% in the per-protocol analysis.^16^ By comparison, the first shock conversion success rate of the Jewel was 88.9%. Similar to commercially available WCDs, the Jewel employs the BTE waveform for shock delivery.^17,18^ This waveform design has previously been demonstrated to be highly effective at termination of VT/VF with shorter time to first post-shock sinus beat.^19–24^ Furthermore, lower-energy biphasic waveform shocks are safer and have demonstrated equivalent or higher success in terminating VF when compared with monophasic waveform shocks delivering escalating energy.^19,20^ An important difference between the Jewel and existing WCDs is the defibrillation delivery interface. Due to the adhesive-based design of the Jewel, the shock is delivered via a hydrogel interface that maintains uniform and continuous contact with the skin. On the other hand, the LifeVest and ASSURE have gel-extruding electrodes which are activated when shock delivery is anticipated. Uneven distribution of the gel increases the risk of skin burns as a consequence of shock delivery.^25^ By comparison, no skin burns or adverse events were reported in this study. The safety and effectiveness of defibrillation with the Jewel P-WCD in the intended use population was studied separately in the Jewel IDE Study and met all primary and secondary endpoints.^11^

Unlike studies of other commercially available WCDs, this study evaluated defibrillation effectiveness using the form factor of the device intended for commercial use. By comparison, in the ACE-CONVERT study of the Kestra ASSURE WCD, commercially available adhesive electrodes were used to deliver the shock rather than the WCD device itself.^16^ In the subsequent ACE-DETECT study of this WCD, episodes of VT/VF were terminated by pre-existing ICDs rather than the ASSURE WCD.^26^

### Limitations

While the sample size of this study was small and limited to a single centre within the EU, it is comparable to previously published efficacy studies of WCD technologies.^13–15^ Additionally, some patients who would be eligible for a WCD (i.e. individuals with LVEF < 20% or with recent amiodarone use) were excluded; however, all patients enrolled in this study had ICDs and therefore were previously determined to be at risk for SCA. The subgroups excluded in this study were subsequently studied in the Jewel IDE Study which demonstrated the safety and effectiveness of the Jewel P-WCD in the intended use population (NCT05201495).^11^ Overall, this study was designed to demonstrate the performance of the Jewel device at VT/VF termination; therefore, the inclusion and exclusion criteria used in the study design were sufficient and pragmatic. Additionally, while this study evaluated the safety and effectiveness of a single shock at 150 J, the Jewel P-WCD is capable of delivering a salvo of five shocks with subsequent shocks after the first delivered at higher energy (162 J).^11^ For ethical reasons and to ensure patient safety by terminating VT/VF as rapidly as possible, the true overall defibrillation effectiveness of the Jewel P-WCD designed for commercial use, which was used in the Jewel IDE Study, was not determined. Finally, the Jewel algorithm, which uses longer fixed intervals of time to classify and confirm potential shockable rhythms, was not evaluated in this study but was evaluated in the Jewel IDE Study.^11^ Operator-controlled shock delivery was enabled to allow for rapid defibrillation at any time after operator confirmation of VT/VF to optimize for participant safety.

### Clinical implications

This first-in-human evaluation of the Jewel P-WCD demonstrated the feasibility of a novel, adhesive-based WCD device in effectively terminating VT/VF with a single shock. There were no adverse events, including burns, reported during this study. The Jewel, which was designed to optimize wearability and convenience and thus improve compliance, has the potential to offer improved protection for patients at high risk of SCA.

## Data Availability

The data that support the findings of this study are available from the corresponding author upon reasonable request.
